# Persistence of Functional Protein Domains in Mycoplasma Species and their Role in Host Specificity and Synthetic Minimal Life

**DOI:** 10.3389/fcimb.2017.00031

**Published:** 2017-02-07

**Authors:** Tjerko Kamminga, Jasper J. Koehorst, Paul Vermeij, Simen-Jan Slagman, Vitor A. P. Martins dos Santos, Jetta J. E. Bijlsma, Peter J. Schaap

**Affiliations:** ^1^Laboratory of Systems and Synthetic Biology, Department of Agrotechnology and Food Sciences, Wageningen University and ResearchWageningen, Netherlands; ^2^Bioprocess Technology and Support, MSD Animal HealthBoxmeer, Netherlands; ^3^Discovery and Technology, MSD Animal HealthBoxmeer, Netherlands

**Keywords:** mycoplasma, mollicutes, protein domains, genome comparison, host specificity, niche specificity, minimal genome, protein metabolism

## Abstract

Mycoplasmas are the smallest self-replicating organisms and obligate parasites of a specific vertebrate host. An in-depth analysis of the functional capabilities of mycoplasma species is fundamental to understand how some of simplest forms of life on Earth succeeded in subverting complex hosts with highly sophisticated immune systems. In this study we present a genome-scale comparison, focused on identification of functional protein domains, of 80 publically available mycoplasma genomes which were consistently re-annotated using a standardized annotation pipeline embedded in a semantic framework to keep track of the data provenance. We examined the pan- and core-domainome and studied predicted functional capability in relation to host specificity and phylogenetic distance. We show that the pan- and core-domainome of mycoplasma species is closed. A comparison with the proteome of the “minimal” synthetic bacterium JCVI-Syn3.0 allowed us to classify domains and proteins essential for minimal life. Many of those essential protein domains, essential Domains of Unknown Function (DUFs) and essential hypothetical proteins are not persistent across mycoplasma genomes suggesting that mycoplasma species support alternative domain configurations that bypass their essentiality. Based on the protein domain composition, we could separate mycoplasma species infecting blood and tissue. For selected genomes of tissue infecting mycoplasmas, we could also predict whether the host is ruminant, pig or human. Functionally closely related mycoplasma species, which have a highly similar protein domain repertoire, but different hosts could not be separated. This study provides a concise overview of the functional capabilities of mycoplasma species, which can be used as a basis to further understand host-pathogen interaction or to design synthetic minimal life.

## Introduction

Mycoplasmas have evolved from a common gram-positive ancestor (Razin and Yogev, 1998) and the evolutionary path of genome reduction has led to an obligatory parasitic lifestyle which presumably has selected for those bacteria that best manipulate their hosts and make optimal use of their specific niche with a minimal set of genes. The mechanisms needed by these bacteria to survive in a vertebrate host, however, are not completely understood (Rosengarten et al., 2000). Research into infectious mechanisms used by mycoplasma species has been focused on identification of adhesive molecules (Rottem, 2003), lipoproteins (Browning et al., 2011), molecular mechanisms used to vary the composition of the surface of the bacterial membrane (Razin and Yogev, 1998) and production of oxidizing components (e.g., hydrogen peroxide and hydrogen sulfide) which cause damage to the host (Vilei and Frey, 2001; Großhennig et al., 2015). While these studies provide insight into the mechanisms used by mycoplasmas to infect the host they do not explain why a mycoplasma species is specific for its host. Besides being important pathogens, mycoplasma species have also been extensively studied because their gene set is expected to be close to the minimal amount of genes needed to sustain life (Gil et al., 2004). Recently, a major hallmark was achieved by publication of an engineered mycoplasma with a synthetic minimal genome of 473 genes based on the genome of *Mycoplasma mycoides* subsp. *capri* (Gibson et al., 2010; Hutchison et al., 2016) providing a benchmark for genome comparison studies aimed at determining gene essentiality.

Advancements in genome sequencing techniques led to the availability of a multitude of genomes from mycoplasma species. With this wealth of sequencing data, it is possible to study the complete repertoire of genes for a bacterial species, the pan-genome. Rouli et al. (2015) observed that bacterial species that have adopted an allopatric lifestyle in specific hosts, tend to have a closed pan-genome. In recent comparative genomics studies for mycoplasma and haemoplasma species, a sub-group within the mycoplasma genus, the pan-genome was reported to be open (Liu et al., 2012; Guimaraes et al., 2014). Here we present a genome-scale comparison of mycoplasma species at the functional level of protein domains. Proteins are the main working machinery of the cell and consist of functional domains, which are stable structurally independent and genetically mobile units. A protein function can thus be precisely described by taking into account the specific domain composition architecture (Koehorst et al., 2016b). Studying protein domain presence, instead of gene sequence similarity, allows for comparison of domain promiscuity, and expansion and domain architecture variability. In a recent study, this approach was used for comparison of 121 *Streptococcus* strains based on the protein domain composition of these strains (Saccenti et al., 2015) and the authors were able to capture metabolic flexibility within *Streptococcus* through the identification of differences in core metabolic pathways between pathogenic and non-pathogenic strains. By analyzing functional capability based on protein domains, we gain insight in functional flexibility of mycoplasma species and we hypothesized that this will allow us to capture functional differences between mycoplasma species explaining adaptation to a host or niche. This strategy is supported by the recent finding that for *Mycoplasma pneumonia* gene essentiality should be studied on the level of domains and not on the level of genes (Lluch-senar et al., 2015). All protein domains found in a species make up the pan-domainome of a species (Kuznetsov et al., 2006), containing core, accessory, and unique domains.

We performed a de novo annotation of 80 publically available mycoplasma genomes and included in this analysis the synthetic minimal genome variant of *M. mycoides* subsp. *capri* using a standardized pipeline for prokaryotic genomes focused on identification of protein domains. We determined the composition and size of the core- and pan-domainome of distinct mycoplasma species and of the complete mycoplasma genus. Incorporation of the synthetic minimal variant in the comparison allowed us to analyze the overlap between protein domains in the core domainome of mycoplasma species vs. the synthetic minimal bacterium to pinpoint functions essential for minimal life.

## Methods

### Genome retrieval and data handling

In total 65 complete and 15 draft mycoplasma genomes (Table S1) were obtained from the NCBI database on the 25th of August 2015 using the “rsync” interface. The dataset contained information from 34 mycoplasma species. For 20 species a single genome sequence was available while for the other 14 species multiple genomes were available (2–12 genomes per species). For 6 species only a draft genome sequence was available. Genome sizes range from 0.58 Mbp for *M. genitalium* to 1.36 Mbp for *M. penetrans*. Genome sequences were retrieved in FASTA format and were used as input for an in-house prokaryotic annotation platform (SAPP; Koehorst et al., 2016a). *Bacillus subtilis* strain 168 (NC_000964) (Weisburg et al., 1989) was used as outlier/common ancestor. Briefly, the SAPP platform consists of sets of modules required for genome annotation of prokaryotes. Different modules can be selected for analysis and results and metadata are directly stored in a graph-database using the RDF (Resource Description Framework) data model. Originally deposited genome annotations were obtained directly from the NCBI in GenBank format and converted into RDF. For three draft genomes no reference annotation was available (accession numbers: NZ_ANIV00000000, NZ_ANAB00000000, and NX_ANAA00000000).

### Genome re-annotation using SAPP

Gene prediction was performed using Prodigal version 2.6.2 (Hyatt et al., 2010) with codon table 4 (The Mold, Protozoan, and Coelenterate Mitochondrial Code and the Mycoplasma/Spiroplasma Code). Proteins were analyzed using InterProScan version 5.4-47.0 (Jones et al., 2014) with the complete set of applications enabled (TIGRFAM, PIRSF, ProDom, SMART, PROSITE Profiles&Pattern, HAMAP, PfamA, PRINTS, SUPERFAMILY, Coils and Gene3D). Protein domain information and other relevant information (GO terms, EC#'s) obtained from InterProScan were directly stored in the graph-database. For querying results a SPARQL endpoint was set-up on a local server using Blazegraph Workbench v2.1.0. The annotated genomes were uploaded in RDF format using the Blazegraph Webinterface and query results were obtained in R using RCurl (Temple Lang, 2015) and SPARQL (Van Hage, 2013).

### Phylogenetic analysis of mycoplasma genomes

16S rRNA sequences were obtained from the ARB-SILVA database (Quast et al., 2013) (Table S2). When available, sequences from the “all species living tree” project were used. For the synthetic JCVI-Syn3.0, the 16S rRNA sequence of the parental *M. mycoides* subsp. *capri* PG3 was used. 16S rRNA sequences were aligned with Clustal Omega (version 1.2.1). MEGA (version 7.0.14) was used to create a phylogenetic tree (maximum likelihood method with 500x bootstrapping). Archaeopteryx (version 0.9901) was used to visualize the tree and root the tree using *B. subtilis* as outlier. The phylogenetic tree was read into R and analyzed using the R package “ape” (Paradis et al., 2004). Comparison of the phylogenetic tree to the protein domain tree was done using the R package “dendextend” (Galili, 2015).

### Analysis of core- and pan-domainome

The total domain composition of each genome was obtained using SPARQL queries. Only domains which were assigned with an e-value of <1E^−07^ were taken into account. In R, a matrix was created with all genomes and their domain composition in binary format, meaning that in this analysis only domain presence was considered. Clustering of species based on the presence/absence matrix was done using the function “hclust” in R; distances were calculated using the “Manhattan” distance. The R-package “micropan” (Snipen et al., 2009) was used to analyze the pan- and core-domainome of species from which five or more genomes were available and the same approach was used to analyze the complete mycoplasma database. To analyze how the amount of genomes sequenced affects the size of the pan- and core-domainome, a 10 times random sampling was done from the presence/absence domain matrix using sample sizes ranging from 1 to 80 genome sequences. The range of model complexities considered (k-range) was 3–5. Estimated core- and pan-domainome sizes were obtained using micropan; true core- and pan-domainome sizes were directly calculated from the sample set. Further analysis of differences between species was done using principal component analysis (PCA). Loading scores obtained with PCA were used to identify domains that contribute highly to group separation. To identify domains present in haemoplasma species that contribute highly to separation of this cluster from the other mycoplasma clusters a loadings score >0.02 was used. To identify domains that contribute highly to separation of the pneumoniae cluster and the spiroplasma/hominis cluster, cut-off values for the loading score of >0.05 and <–0.05 were used, respectively. Proteins with a metabolic function were extracted from the genome-scale metabolic model of *M. pneumonia* (Wodke et al., 2013) and extended with InterProScan domain annotations.

### Analysis of orthologous proteins

A SPARQL query was used to generate a protein FASTA file using all mycoplasma genomes (JCVI-Syn3.0 was not taken into account). An all-against-all BLASTP (Wolf and Koonin, 2012) was performed of the mycoplasma proteins using an e-value cut-off of 1E^−05^ and a maximum target sequence of 10^5^. The BLAST file created was used to find orthologous proteins with orthAgogue (Ekseth et al., 2014) excluding protein pairs with an overlap below 50%. Clustering of orthologous proteins was done using MCL (Enright et al., 2002) setting 1.5 as main inflation. With a SPARQL query the domain composition of all orthologous proteins was obtained based on InterPro identifiers.

### Clustering of hypothetical mycoplasma proteins

Hypothetical proteins (domain-less proteins) were obtained from the mycoplasma genomes using a SPARQL query. Orthologous protein clusters containing these hypothetical proteins were obtained from the list of orthologous protein clusters. Persistence of these orthologous clusters was determined in the complete set of genomes used, JCVI-Syn3.0 and *M. mycoides* subsp. *capri LC*. Haemoplasma species were excluded from this analysis.

### Prediction of host/niche specific domains

K-nearest neighbor and random forest classification (Breiman, 2001) were used to classify mycoplasma species based on host or niche specificity and to identify domains important for classification. A binary domain presence/absence matrix was used as input. The R-package “class” was used to perform k-nearest neighbor (k-nn) classification (Venables and Ripley, 2002) and the R package “randomForest” (Liaw and Wiener, 2002) was used for random forest classification. 500 trees were built for each classification with random forest. Domains important for classification were found based on the mean decrease in node impurity (Gini index). Information from 26 mycoplasma genomes was used for the final niche classification and from 22 genomes for the final host classification (Tables S10, S11). K-nn classification for the niche dataset was done with a k-value of 5, 19 mycoplasma species in the training set and 7 mycoplasma species in the test set (4 infecting multiple tissue types and 3 infecting strictly respiratory tissue). For the host classification a k-value of 4 was used, 16 mycoplasma species in the training set and 6 mycoplasma species were used in the test set (3 species infecting ruminants, 1 species infecting pig and 2 species infecting humans).

## Results

### Re-annotation of mycoplasma genomes

The quality of the structural and functional annotation of publically available genomes can vary. In order to get for all studied genomes an up-to-date set of annotated genes and to minimize the risk of false discoveries resulting from methodological inconsistencies, the 80 publicly available mycoplasma genomes (Table S1) were re-annotated using a standardized set of algorithms (Koehorst et al., 2016a). Mycoplasma genomes have a low GC content and thus accuracies of the various original gene prediction methods applied were expected to be high (Tripp et al., 2015). Nevertheless, on average 3.7% more genes were found after re-annotation with the most recent version of prodigal (Hyatt et al., 2010). Consequently, the total number of proteins found in the re-annotated genomes was also higher (Table S3).

### Mycoplasma proteome and predicted pan- and core-domainome

**Figure 1:**
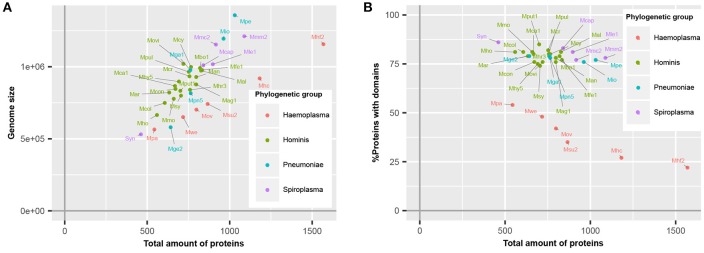
**Mycoplasma proteome specification. (A)** Correlation between genome size and total amount of proteins. **(B)** Ratio of proteins covered by protein domains. Mag, *M. agalactiae*; Mal, *M. alligatoris*; Man, *M. anatis*; Mca, *M. canis*; Mcol, *M. columbinum*; Mge, *M. genitalium*; Mio, *M. iowae*; Mmc, *M. mycoides capri*; Movi, *M. ovipneumoniae*; Mpn, *M. pneumoniae*; Mar, *M. arthritidis*; Mbo, *M. bovis*; Mca, *M. capricolum* subsp. *capricolum*; Mcon, *M. conjunctivae*; Mcr, *M. crocodyli*; Mcy, *M. cynos*; Mfe, *M. fermentans*; Mga, *M. gallisepticum*; Mhc, *M. haemocanis*; Mhf, *M. haemofelis*; Mho, *M. hominis*; Mhy, *M. hyopneumoniae*; Mhr, *M. hyorhinis*; Mle, *M. leachii*; Mmo, *M. mobile*; Mmm, *M. mycoides* subsp. *mycoides*; Mov, *M. ovis*; Mpa, *M. parvum*; Mpe, *M. penetrans*; Mpul, *M. pulmonis*; Mput, *M. putrefaciens*; Msu, *M. suis*; Msy, *M. synoviae*; Mwe, *M. wenyonii*; Syn, JCVI-Syn3.0. Numbers relate to strains (Table S4).

Haemoplasma species, which specifically infect blood, have a higher number of predicted proteins relative to their genome size (Figure 1A), corresponding with a lower average CDS length (Guimaraes et al., 2011). This difference is caused by the presence of a large repertoire of proteins with a relatively short CDS length, which are part of paralogous gene families (do Nascimento et al., 2012). To survive in their specific niche, haemoplasma species can express these proteins and generate variability of proteins at the cell surface to prevent detection by the immune system of the host (Citti and Blanchard, 2013). Besides the haemoplasma species, a high amount of predicted proteins relative to the genome size was also found in *M. genitalium* G37 and JCVI-Syn3.0. Approximately 80% of the mycoplasma species proteins contained functional domains (Figure 1B). This percentage is similar to the average match percentage found if the whole UniProtKB is analyzed using InterProScan (Hunter et al., 2012). The *M. mycoides* based JCVI-syn3.0 synthetic genome contained the highest percentage of proteins with a recognizable domain (86%), ~9% more than the parental template genome. Haemoplasma species were the notable exceptions, which despite their normal genome size, contained a significantly lower percentage of proteins with recognizable domains (22–54%). This difference occurs because the aforementioned variable surface proteins do not contain recognizable domains. As a direct result of the re-annotation strategy the total amount of unique functional domains per species increased with 0.8% on average.

**Figure 2:**
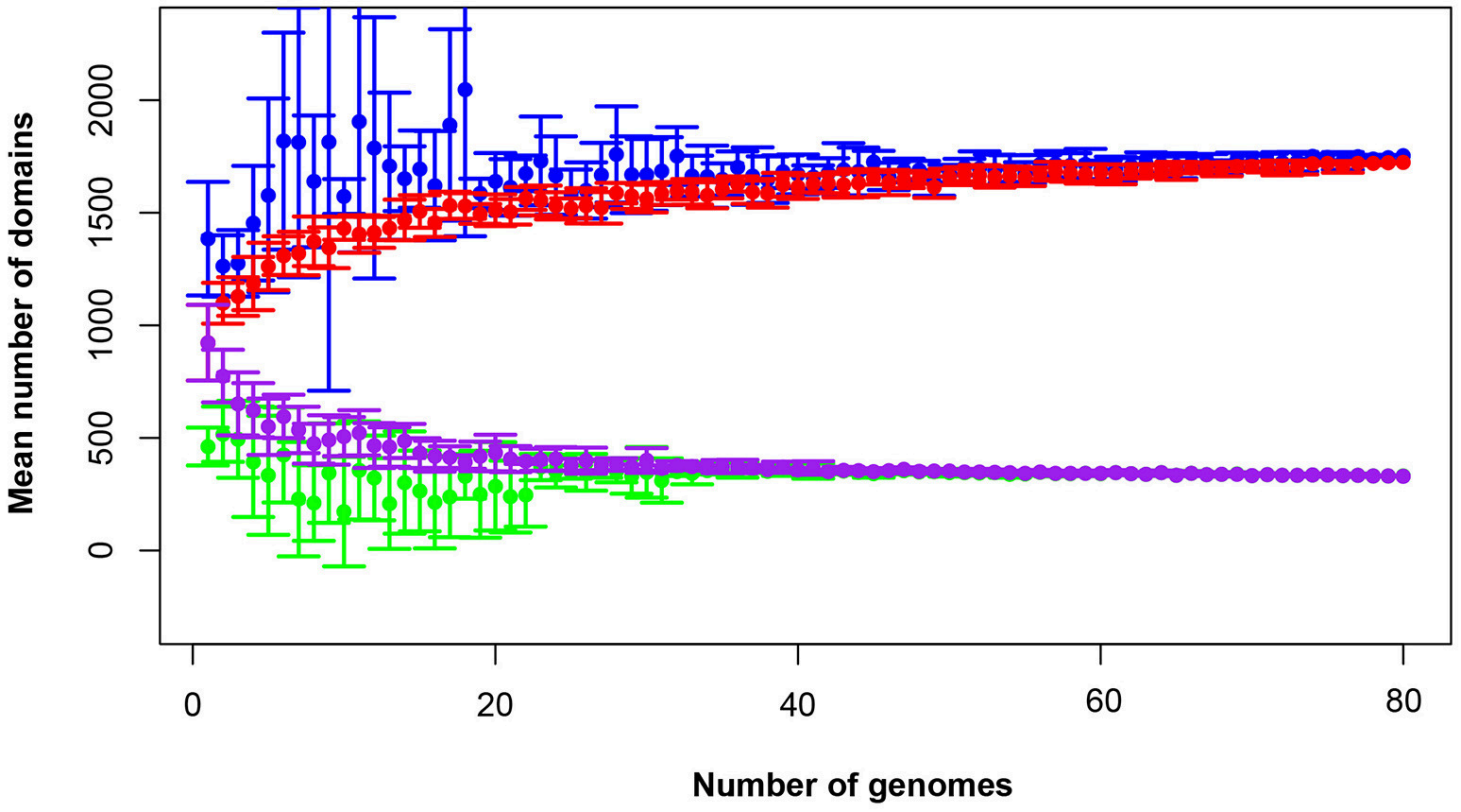
**The mycoplasma pan-domainome is closed**. True and estimated core- and pan-domainome sizes were calculated using an iterative process (*n* = 10) in which a fixed number of genomes was randomly selected from the binary domain matrix. Estimated values were calculated using the R package MicroPan and true values were directly calculated from the input. Estimated pan-domainome (blue); true pan-domainome (red); estimated core-domainome (green) and true core-domainome (purple).

The total pan-domainome consisted of 1737 domains, the core domainome consisted of 335 domains and the core-to-pan ratio was 19.3%. Analysis of the pan-domainome for species from which 5 or more genomes (*M. pneumoniae, M. gallisepticum, M. hyopneumoniae*, and *M. genitalium*) were available using Micropan (2- or 3-component system, Snipen et al., 2009) showed that the pan-domainome was closed (alpha > 1). A closed pan-domainome was also observed for the genus (9 component system, alpha > 1) taking into account all 80 mycoplasma genomes (Figure 2).

### Functional classification of mycoplasma species

**Figure 3:**
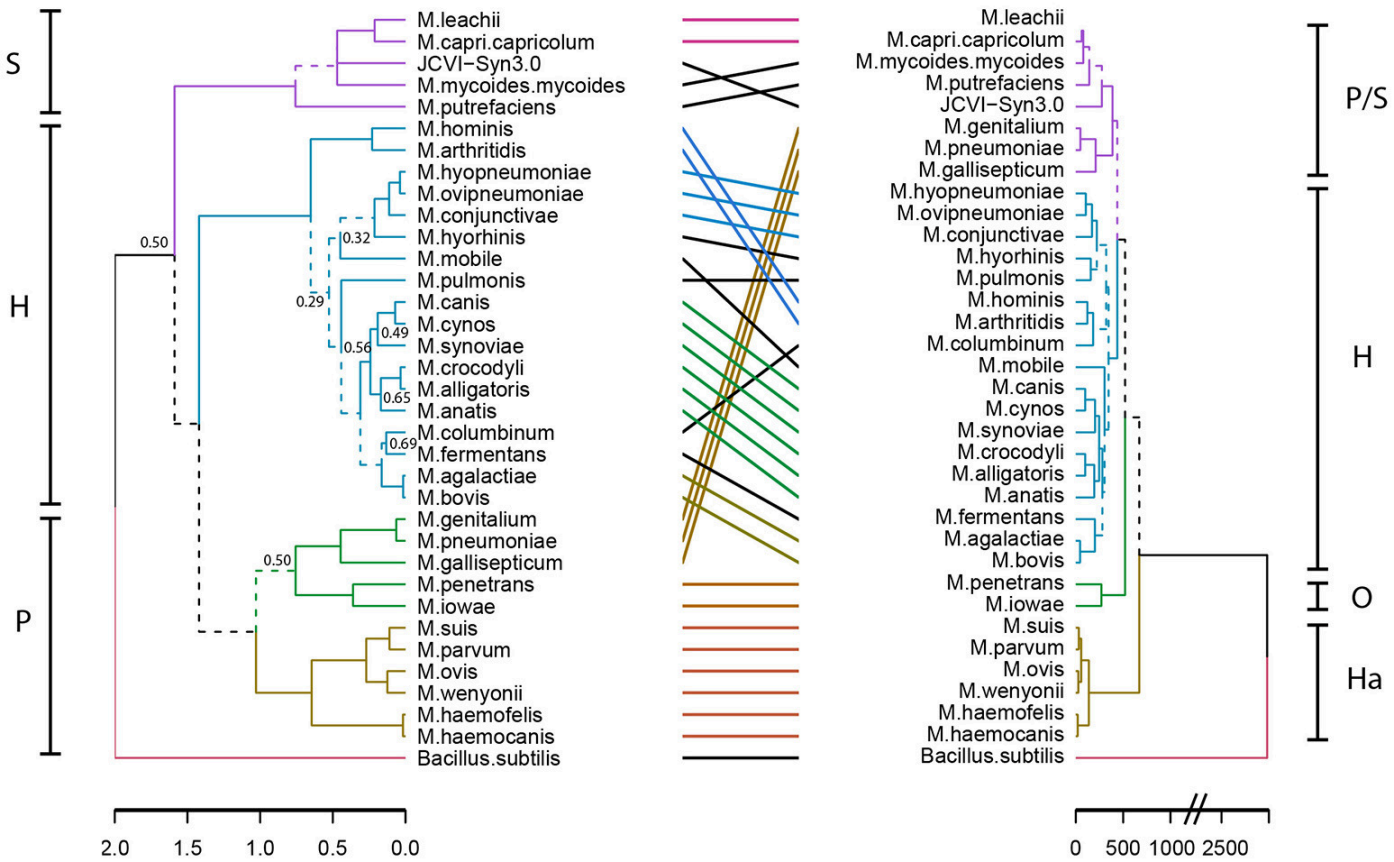
**Niche-driven functional evolution**. Accelerated functional evolution causes separation of haemoplasma species and several other mycoplasma species when phylogenetic clusters are compared to functional clusters. Dashed lines indicate distinct branches. **Left:** Standard phylogenetic tree using 16S rRNA (maximum likelihood, 500x bootstrapped, see Table S2 for strains and sequences which were used). Only bootstrapping values <0.7 are shown, the phylogenetic tree with all bootstrapping values is shown in Figure S2. **Right:** functional clustering based on Manhattan distance calculated from the presence/absence matrix of domains. Groups indicated are: S, Spiroplasma; H, Hominis; P, Pneumoniae; Ha, Haemoplasma; and O, Other.

To gain insight into a possible functional differentiation of mycoplasma species as a result of specific host co-evolution, we clustered mycoplasma species based on a presence/absence domain matrix and compared domain repertoire clustering with clustering based on 16S rRNA sequences (Figure 3). In the domain based functional tree, the monophyletic pneumonia cluster separated into three separate functional clusters. One of these separate clusters contains the haemoplasma species, which have a relatively low number of protein domains (Figure S1 and Table S4). *M. penetrans* and *M. iowae* form a second functional cluster; these species have a relatively high number of functional domains when compared to other species in the pneumonia 16S-phylogenetic group. The remaining species in this 16S-phylogenetic group are closely related to the spiroplasma cluster in the functional tree. The hominis 16S-phylogenetic cluster was completely maintained in the protein domain tree but compared to the 16S tree there were some re-arrangements, which can partly be explained by low significance in the assignment of branches in the 16S phylogenetic tree. Notable changes are: *M*. *hominis* and *M. arthritidis* clustered with *M. columbinum* and *M. pulmonis* clustered with *M. hyorhinis*. We did not observe a functional clustering based on host.

### Functional differentiation of haemoplasma species

**Figure 4:**
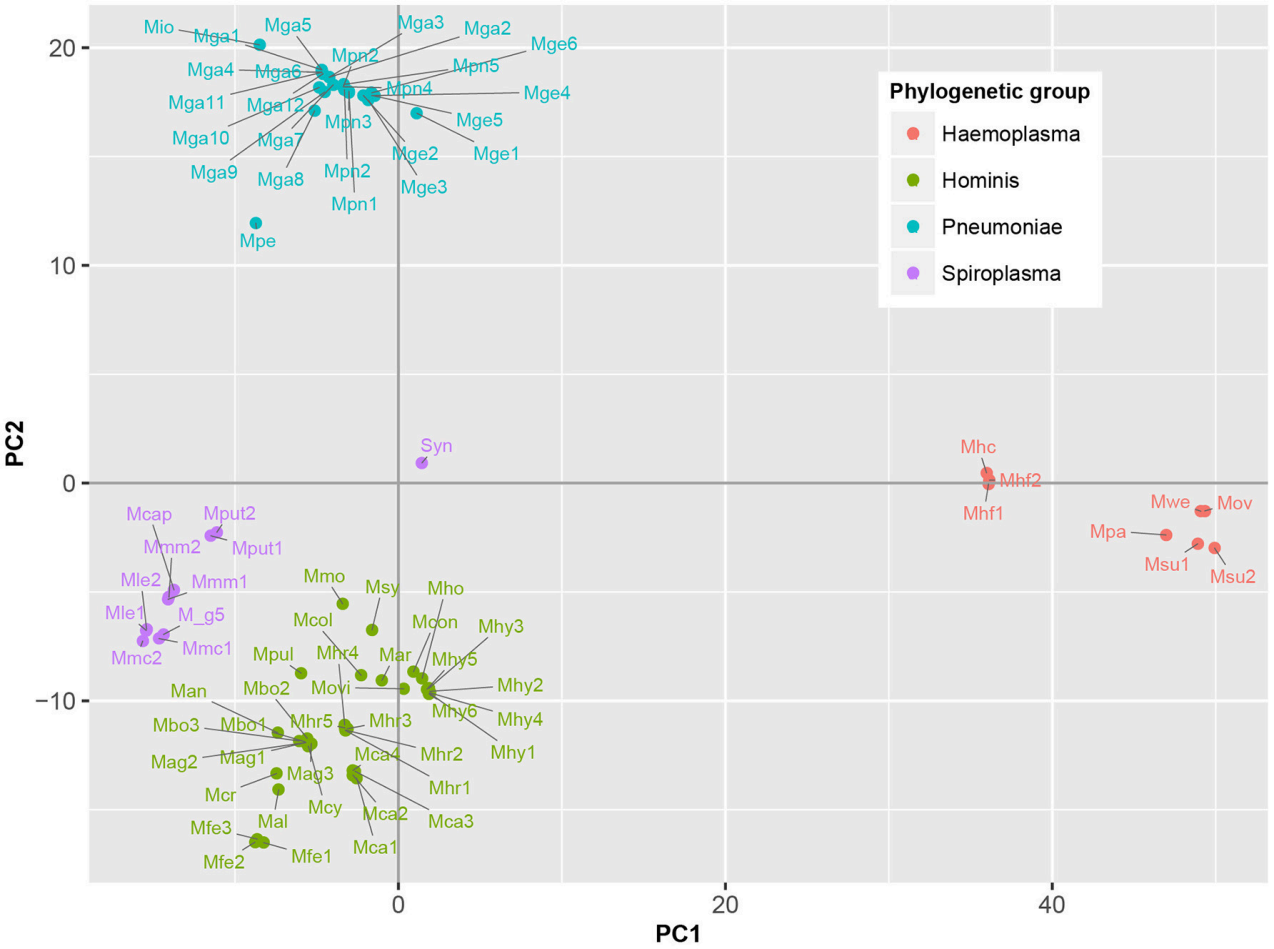
**Functional differentiation of mycoplasma species**. Score plot is shown of principal component analysis done on the presence/absence matrix of the 80 mycoplasma strains and the synthetic bacterium JCVI-Syn3.0. Main phylogenetic groups are color coded. Note the separation of mycoplasma species infecting blood and tissue. Mag, *M. agalactiae*; Mal, *M. alligatoris*; Man, *M. anatis*; Mca, *M. canis*; Mcol, *M. columbinum*; M_g5, *M. g5847*, Mge, *M. genitalium*; Mio, *M. iowae*; Mmc, *M. mycoides capri*; Movi, *M. ovipneumoniae*; Mpn, *M. pneumoniae*; Mar, *M. arthritidis*; Mbo, *M. bovis*; Mca, *M. capricolum* subsp. *capricolum*; Mcon, *M. conjunctivae*; Mcr, *M. crocodyli*; Mcy, *M. cynos*; Mfe, *M. fermentans*; Mga, *M. gallisepticum*; Mhc, *M. haemocanis*; Mhf, *M. haemofelis*; Mho, *M. hominis*; Mhy, *M. hyopneumoniae*; Mhr, *M. hyorhinis*; Mle, *M. leachii*; Mmo, *M. mobile*; Mmm, *M. mycoides* subsp. *mycoides*; Mov, *M. ovis*; Mpa, *M. parvum*; Mpe, *M. penetrans*; Mpul, *M. pulmonis*; Mput, *M. putrefaciens*; Msu, *M. suis*; Msy, *M. synoviae*; Mwe, *M. wenyonii*; Syn, JCVI-Syn3.0. Numbers relate to strains (Table S4).

To determine which domains were important for separation of haemoplasma from mycoplasma species infecting tissue, we used principal component analysis (Figure 4). Based on the loading scores for the first and second principal component we could assess which domains contributed to group separation. Haemoplasma species were separated from the other mycoplasma species along the first principal component. We identified 30 domains in haemoplasma species that mainly contributed to separation of this cluster (Table 1 and Table S5) and 400 domains present in the tissue infecting mycoplasma species that mainly contributed to separation of this cluster from the haemoplasma species cluster. Domains present in the haemoplasma species that contributed to group separation were ABC transporter domains for iron or vitamin B12. Multiple domains were found related to functional enzymes in purine metabolism (GMP synthase, IMP dehydrogenase, adenylosuccinate synthase) or L-aspartate metabolism (fumarate lyase family domains, part of adenylosuccinate lyase) which provides a precursor for purine metabolism (Santos et al., 2011). The presence of GMP synthase domains may provide the haemoplasma with the option to produce all purine bases from hypoxanthine which is present in blood (Guimaraes et al., 2011). An alternative function for these GMP synthase domains could be the production of glutamate which is present in a low concentration in blood (McMenamy et al., 1957). Three domains related to superoxide dismutase activity were also found, a function, which could provide protection when radicals are present in blood.

**Table 1:** **Top 10 domains responsible for separation of mycoplasma functional clusters**.

**Enriched in haemoplasma^a^**		**Enriched in hominis/spiroplasma^b^**		**Enriched in pneumoniae^c^**	
**ID^d^**	**InterPro description^e^**	**ID^d^**	**InterPro description^e^**	**ID^d^**	**InterPro description^e^**
IPR026023	Ribonucleotide reductase small subunit, prokaryotic	IPR029048	Heat shock protein 70kD, C-terminal domain	IPR002606	Riboflavin kinase, bacterial
IPR029022	ABC transporter, BtuC-like	IPR013826	DNA topoisomerase, type IA, central region, subdomain 3	IPR003526	2-C-methyl-D-erythritol 2,4-cyclodiphosphate synthase
IPR000522	ABC transporter, permease protein	IPR004398	RNA methyltransferase, RsmD	IPR006660	Arsenate reductase-like
IPR001674	GMP synthase, C-terminal	IPR003442	tRNA threonylcarbamoyl adenosine modification protein TsaE	IPR011631	Protein of unknown function DUF1600
IPR004837	Sodium/calcium exchanger membrane region	IPR006667	SLC41 divalent cation transporters, integral membrane domain	IPR023344	Uncharacterized domain MG237, C-terminal
IPR001670	Alcohol dehydrogenase, iron-type	IPR006668	Magnesium transporter, MgtE intracellular domain	IPR015271	Protein of unknown function DUF1951
IPR001093	IMP dehydrogenase/GMP reductase	IPR016947	Bacteriophage gamma, gammalsu0035	IPR013825	DNA topoisomerase, type IA, central region, subdomain 2
IPR019065	Restriction endonuclease, type II, NgoFVII	IPR000748	Pseudouridine synthase, RsuA/RluB/E/F	IPR012760	RNA polymerase sigma factor RpoD, C-terminal
IPR020471	Aldo/keto reductase subgroup	IPR001525	C-5 cytosine methyltransferase	IPR001844	Chaperonin Cpn60
IPR023210	NADP-dependent oxidoreductase domain	IPR003370	Chromate transporter	IPR002423	Chaperonin Cpn60/TCP-1

a *Domains enriched in haemoplasma functional cluster*.

b *Domains enriched in hominis/spiroplasma functional cluster*.

c *Domains enriched in pneumoniae functional cluster*.

d *InterPro Identifier*.

e *Domain description obtained from InterProScan*.

### Functional differentiation between the hominis/spiroplasma and pneumoniae groups

Along the second principal component the hominis and spiroplasma clusters were separated from the pneumoniae cluster. We found 43 domains present in the hominis/spiroplasma clusters that mainly contributed to separation from the pneumoniae cluster vs. 71 in the pneumoniae cluster that mainly contributed to separation from the hominis/spiroplasma cluster (Table 1 and Table S5). In the hominis/spiroplasma cluster there was an increased presence of domains related to transport of magnesium and other divalent cations and also an increased capacity for chromate transport. Metals are important co-factors and increased chromate transport capability possibly results in increased chromate resistance as observed in *B. subtilis* (Díaz-Magaña et al., 2009). Functionalities of other domains important to separate the hominis/spiroplasma cluster from the pneumoniae cluster were related to DNA/RNA modification, protein/peptide degradation and phosphopentomutase activity. The latter enzyme links nucleotide synthesis to the pentose phosphate pathway (PPP) (Pollack et al., 1997) and provides mycoplasma with the option to produce nucleotides from the purine/pyrimidine bases or alternatively to degrade nucleotides via the PPP and glycolysis. In the set of domains that mainly contributed to separation of the pneumonia cluster from the hominis/spiroplasma cluster, a functional domain related to NAD kinase activity, needed for the production of NADP^+^, was found. Another domain was found linked to activity in the non-mevalonate pathway of isoprenoid synthesis: 2-C-methyl-D-erythritol 2,4-cyclodiphosphate synthase. Activity of this pathway was shown for *M. penetrans* and *M. gallisepticum* (Eberl et al., 2004) and might reduce the need to obtain isoprenoid precursors from the host. There was an increased presence of a domain related to thioredoxin-disulfide reductase activity which produces reduced thioredoxin needed for the production of deoxyribonucleotides and is important for protection against oxidative stress (Ben-Menachem et al., 1997). The separation of mycoplasma species based on protein domain composition provided a concise overview of the functional differences between mycoplasma species.

### Persistence of protein domains and of orthologous proteins

**Figure 5:**
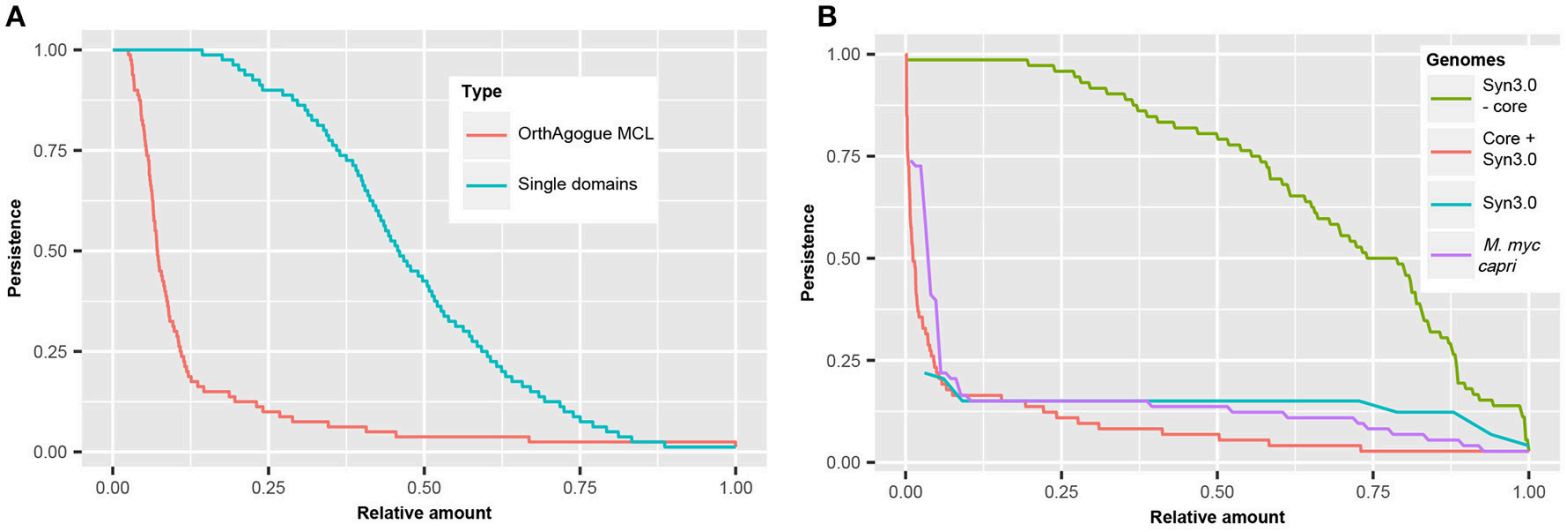
**Persistence of (essential) domains and (essential) hypothetical proteins. (A)** Persistence of orthologous proteins based on sequence similarity (red) and persistence of single domains (blue). **(B)** Persistence of domains present in JCVI but absent from the core of the mycoplasma species infecting tissue (green). Persistence of hypothetical proteins for 72 mycoplasma species including JCVI-Syn3.0 (red), *M. mycoides capri* LC (purple), and JCVI-Syn3.0 (blue).

In order to compare the persistence of protein domains with the persistence of orthologous proteins, the complete set of orthologous proteins in the 80 mycoplasma genomes was determined using a standard bidirectional best hit approach (Wolf and Koonin, 2012) followed by orthology assessment with orthAgogue (Ekseth et al., 2014) and MCL clustering (Enright et al., 2002). We found >5000 clusters of orthologous proteins and examined in how many genomes these orthologous proteins are present (Figure 5A). Only 135 orthologous proteins are conserved amongst all mycoplasma species and we find an average persistence of orthologous proteins of 12.6%. The persistence of protein domains in the pan-domainome was much higher (average of 48.4%, Figure 5A).

### Mycoplasma pan- and core-domainome analysis in relation to JCVI-syn3.0

Clustering of mycoplasma species based on the pan-domainome did not show a correlation with their specific host. To further classify protein domains, we compared the pan- and core-domainome of the mycoplasma genus with the domainome of the minimal synthetic organism JCVI-Syn3.0 (Hutchison et al., 2016) which consisted of 869 domains (Table S7). For the synthetic organism we assumed that all protein domains in this organism were essential. The core domainome consisted of 335 domains, a relatively small amount. This can be explained because we took into account the haemoplasma species that grow in blood and cannot be cultured *ex vivo*. When the core-domainome was calculated for mycoplasma species infecting tissue, a larger size core was obtained of 479 protein domains. From this core 26 domains were not present in JCVI-Syn3.0 (Table S8). Apparently these domains are not essential for growth in a laboratory environment. The remaining 453 core domains show overlap with JCVI-Syn3.0 (Table S8), indicating that these persistent domains are essential for axenic growth in (complex) growth media. Interestingly, the remaining 416 domains in JCVI-Syn3.0 essential for minimal life are not persistent (Figure 5B and Figure S3) suggesting that within the mycoplasma domain landscape many alternative configurations exist that bypass their essentiality.

### Metabolic capability in relation to host specificity

To assess if domains with a non-essential metabolic function determine host specificity, we obtained all domains with a metabolic function not present in the synthetic minimal organism. Domains with a metabolic function were derived based on the genome-scale metabolic model of *M. pneumoniae* (Wodke et al., 2013) supplemented with InterPro annotations. This model contains 145 genes, coding for 145 proteins, and from this set of proteins 359 unique protein domains were obtained. Almost all proteins with a metabolic function were covered with domains (97%). Overall we found 162 domains (33.8% of the total core) with a metabolic function to be present in the core of the tissue infecting mycoplasma species and 197 accessory domains with a metabolic function present in the accessory domainome (pan minus core). In JCVI-Syn3 156 domains from the metabolic core domainome and 140 accessory domains with a metabolic function were present. Thus, 63 domains with a metabolic function were absent in JCVI-Syn3.0 and to assess whether these domains could be involved in host specificity we clustered mycoplasma species infecting tissue based on the presence/absence of these domains but we could not establish a correlation with host specificity (Figure S4).

### Role of hypothetical proteins in host adaptation

Clustering based on the pan-domainome composition or on the metabolic domain complement absent in JCVI-Syn3.0 failed to show a direct link between specific domains and host specificity. We further analyzed if presence or absence of hypothetical proteins could explain host specificity. In our dataset, a protein was annotated as hypothetical when a protein did not contain a protein domain or when a protein contained a domain of unknown function (DUF). In total 58 DUFs were found in the mycoplasma genus, from which only 8 DUFs were present in JCVI-Syn3 (Table S9). There were no DUFs in the core domainome of the complete genus and only 2 DUFs in the core domainome of the tissue infecting mycoplasma species (DUF161 and DUF933). DUF161 is part of a membrane protein with unknown function; DUF933 is suggested to be part of a nucleoprotein complex and could function as a GTP-dependent translation factor. The total amount of DUFs found was too low to analyze a relation with the host and for further classification of hypothetical proteins we compared the complete set of hypothetical proteins in JCVI-Syn3.0 to the complete set of hypothetical proteins in the pan-genome of the mycoplasma species infecting tissue. In total 11,598 hypothetical proteins were found in the tissue infecting mycoplasma species which based on sequence similarity, could be clustered into 1766 orthologous protein clusters. The relative persistence of the hypothetical protein clusters showed a sharp decline with an average persistence of approximately 9% (Figure 5B). The total amount of genes with completely unknown functions in the genome of JCVI-Syn3.0 was only 65 (Hutchison et al., 2016) and we identified just 40 proteins to which no functional domains could be assigned. The persistence of orthologous protein clusters containing these hypothetical proteins was 14% (Figure 5B) which was higher than average. There was, however, conservation of clusters with hypothetical proteins from the spiroplasma phylogenetic group. In line with the finding that not all essential JCVI-Syn3.0 protein domains were persistent, essential hypothetical proteins were also not persistent suggesting that within the mycoplasma genus alternative solutions exist substituting these essential but currently unknown functions. We did not observe a relation with the host on the basis of the clustering of orthologous hypothetical proteins not present in JCVI-Syn3.0 (Figure S5).

### Protein domain composition in relation to host or niche

Clustering based on the complete pan-domainome of mycoplasma, the metabolic domains outside JCVI-syn3.0 as well as the hypothetical orthologous proteins outside JCVI-Syn3.0 did not show a relation with a mycoplasma species specific host. As a final effort, we applied two machine learning approaches: k-nearest neighbor (k-nn) and Random Forest (Chen and Ishwaran, 2012), to classify a mycoplasma species niche or host based on the pan-domainome composition. Both methods could predict with high accuracy whether the niche of a mycoplasma species was blood or tissue confirming the results already found using PCA (supplementary materials and Table S5). When the niche was specified in more detail (Table S6, Niche), the prediction accuracy decreased and species with a unique niche (e.g., *M. mobile* and *M. conjunctivae*) could not be assigned. Classification of mycoplasma growing in blood, strictly in the respiratory tract and in multiple tissue types including lung (Table S6, Niche 2) was possible using Random Forest with 95% prediction accuracy (5% out-of-bag error rate). The domain most important for classification was cell division protein *FtsZ* (IPR000158). This domain was present in many mycoplasma species but absent from *M. canis, M. gallisepticum*, and *M. hyopneumoniae*, which formed for a large part the species infecting the respiratory tract in our dataset. Absence of this specific domain does not mean that a species has no functional *FtsZ*, since there are alternative domain configurations possible (containing e.g., domain IPR003008 and IPR020805). To prevent prediction bias due to differences in the number of genomes available of a certain species, we decided to focus on the mycoplasma species infecting tissue for which we had at least two genomes and limited our search to two genomes per species. Using this smaller selection of genomes, prediction accuracy was higher (96% using the random forest classifier and 71% using k-nn classification) and we again identified the specific *FtsZ* domain (Table 2 and Table S10) as an important domain for niche classification. We also identified a putative DNA-binding domain (IPR009061), present in phenylalanine-tRNA synthetases. In our database this domain was not present in the selected strains of *M. canis, M. hyopneumoniae*, and *M. pneumoniae* which are all present strictly in the respiratory tract. The domain was, however, present in other mycoplasma species identified as strictly present in the respiratory tract: *M*. *cynos, M. gallisepticum*, and *M. mycoides* subsp. *mycoides* SC. Also important for classification was restriction endonuclease, type I domain IPR000055, which was not present in *M. gallisepticum* strains used in our selection and was also absent from the *M. mycoides* subsp. *mycoides* SC strains used in our comparison. There was not a single domain uniquely present in all mycoplasma infecting the respiratory tract and absent from the mycoplasma infecting multiple tissue types.

**Table 2:** **Top 10 domains relevant for niche classification: Strictly respiratory or multiple tissue types**.

**Domain information**		**Abundance (%)^a^**	
**ID^d^**	**InterPro description^e^**	**Respiratory system^b^**	**Multiple^c^**
IPR009061	DNA binding domain, putative	40	100
IPR000055	Restriction endonuclease, type I, HsdS	50	94
IPR022749	N6 adenine-specific DNA methyltransferase, N12 class, N-terminal	20	81
IPR000158	Cell division protein FtsZ	40	100
IPR011701	Major facilitator superfamily	50	88
IPR003798	DNA recombination RmuC	20	75
IPR008280	Tubulin/FtsZ, C-terminal	40	88
IPR002198	Short-chain dehydrogenase/reductase SDR	20	75
IPR011089	Domain of unknown function DUF1524	0	50
IPR005864	ATPase, F0 complex, subunit B, bacterial	60	100

a *Abundance of a protein domain in the specific niche*.

b *Abundance in mycoplasma species with a strictly respiratory niche*.

c *Abundance in mycoplasma species with multiple niches including respiratory*.

d *InterPro Identifier*.

e *Domain description obtained from InterProScan*.

For identification of domains important to classify mycoplasma hosts, we first used the complete diversity in hosts mentioned in Table S6 and obtained a prediction accuracy of <80% using random forest. We decided to use a more focused approach and selected only mycoplasma species growing in tissue for which we had two species per host and two genomes per species. Genomes for cows and goats were pooled into a ruminants group. With this grouping, we could accurately predict (83% accuracy with k-nn classification and 100% with random forest) if a mycoplasma species from the selected genomes infects a pig, ruminant, or human. The most discriminatory domains identified from the random forest analysis (Table 3 and Table S11) related to peptidase functions (IPR000668, IPR005151, and IPR029045). A phosphodiesterase domain (IPR024654 and related family IPR000979) was found to be important for host differentiation, this domain only occurs in the human pathogens taken into account. A *RmlC*-like jelly roll fold domain (IPR014710), which is related to mannose/myo-inositol metabolism, was identified in the pig and ruminant species but was absent from species that infect humans. Two domains of unknown function were found: DUF2714 and DUF285 (IPR021222 and IPR005046). The DUF285 domain has probably been exchanged between ruminant species via horizontal gene transfer (Nouvel et al., 2010). Several domains related to proteins expressed at the bacterial surface were found (IPR011889 and IPR027593). A glycine cleavage domain was found (IPR002930) which was absent from the selected mycoplasma species infecting humans. Using the Random Forest prediction, on specific species groups, we have identified a number of protein domains which could relate to host specificity.

**Table 3:** **Top 10 domains relevant for host classification: Ruminants, pigs or humans**.

**Domain information**		**Abundance (%)^a^**		
**ID^e^**	**InterPro description^f^**	**Ruminants^b^**	**Pigs^c^**	**Humans^d^**
IPR000668	Peptidase C1A, papain C-terminal	100	0	0
IPR005151	Tail specific protease	100	0	0
IPR000979	Phosphodiesterase MJ0936/Vps29	0	0	100
IPR014710	RmlC-like jelly roll fold	100	100	0
IPR021222	Protein of unknown function DUF2714	100	100	0
IPR005046	Protein of unknown function DUF285	100	0	0
IPR002931	Transglutaminase-like	100	0	0
IPR002930	Glycine cleavage H-protein	100	100	0
IPR011889	Bacterial surface protein 26-residue repeat	92	0	0
IPR029045	ClpP/crotonase-like domain	100	0	0

a *Abundance of a protein domain in the specific host*.

b *Abundance in ruminant species*.

c *Abundance in pig species*.

d *Abundance in humans*.

e *InterPro Identifier*.

f *Domain description obtained from InterProScan*.

## Discussion

All mycoplasma species have reduced genomes and could be considered minimal organisms. Arguably, the most studied minimal organism to date is *M. pneumoniae*, a human pathogen causing inflammation of lung tissue in humans. From this bacterium we have knowledge of the genome (Dandekar et al., 2000), transcriptome (Güell et al., 2009), proteome (Batisse et al., 2009), metabolome (Yus et al., 2009; Maier et al., 2013) and several regulatory mechanisms including the role of non-coding RNA's (Lloréns-Rico et al., 2016). Interactions with the host have also been extensively studied (Rottem, 2003) but despite of the wealth of information on this minimal organism we still cannot explain why there is a preference for colonization of human lung tissue. Knowing that it is not a simple case of adhesion properties (Krause, 1996), we hypothesized that there is a complex combination of functions that determines a bacterial host or niche. To find these functions, we clustered species based on domain presence to find direct leads and ultimately used a random forest classification algorithm on the complete mycoplasma pan-domainome to find sets of domains that predict the specific host or niche of a mycoplasma species. By considering presence or absence of proteins domains we deviate from the classical approach in which bacterial genomes are compared based on orthologous proteins. We found that the persistence of single domains is higher which indicates that conservation of the structural information in the protein domains is more important than maintaining the gene sequences in which the domains are present. A similar result was recently found in a comparative genomics study of 432 *Pseudomonas* species (Koehorst et al., 2016a), indicating that this could be a trend amongst bacterial species. By using Random Forest classification, we could predict with high accuracy whether a mycoplasma species infects tissue or blood and found metabolic properties in the haemoplasma cluster that could explain why this organism successfully infects blood. Zooming into functional species clusters, the prediction accuracy decreases and it is not possible to predict a host or niche of closely related species such as *M. haemofelis* and *M. haemocanis*, within the haemoplasma group, or *M. agalactiae* and *M. bovis*, within the hominis group. Despite the lower prediction accuracy we were still able to identify differences between mycoplasma species in relation with its specific host or niche if we used larger clusters as was shown for the differentiation of mycoplasma colonizing ruminants, pigs, or humans. To determine the specific role of a signifying protein function (e.g., one of the peptidase functions) in host-pathogen interaction would require additional laboratory studies.

To understand in greater detail which factors determine host or niche specificity, more mycoplasma genomes of species of specific interest could be sequenced. This will provide more detailed information on the variation in the domain composition of this species, increasing the accuracy of host prediction. Further information needed to understand host or niche specificity could also follow from functional annotation of proteins without a protein domain, which make up ~20% of the total proteome of a mycoplasma species. The machine learning approaches applied did not take domain abundance into account as we used the binary domain matrix as input to avoid overfitting. (Dual-)Trancriptomics studies might provide the additional insight needed to explain the interplay between host and pathogen. For example, a recent study on the chicken pathogen *M. gallisepticum* (Pflaum et al., 2015) showed temporal phase variation in the expression of *vlhA* genes during infection. Finally, the strict host specificity for mycoplasma species can be challenged since several mycoplasma species infect a broad range of hosts (e.g., *M. bovis* and *M. mycoides* subsp. *mycoides*) and mycoplasmas normally isolated from animals are sometimes found in humans and vice versa (Huang et al., 2001; Pitcher and Nicholas, 2005). The assumption of strict host specificity for mycoplasma species could be incorrect and mycoplasma may be able to infect a wider range of hosts and ecosystems than previously anticipated (Citti and Blanchard, 2013).

Our finding that the pan-domainome of the mycoplasma genus is closed supports the general expectation that species with an allopatric lifestyle have a lower chance of gaining genes by horizontal gene transfer (HGT). This finding, however, seems to contradict the recent comparative genomics reports on an open pan-genome for mycoplasma species (Liu et al., 2012; Guimaraes et al., 2014). Possible mechanisms that could contribute to the increase of the pan-genome have been described to be: (1) variation in expression and structure of surface antigens, (2) horizontal gene transfer (HGT), (3) genetic drift, and (4) phage attack (Citti and Blanchard, 2013). HGT events between species outside the mycoplasma genus are rare (Sirand-Pugnet et al., 2007) and phage attacks are not common in mycoplasma species (Tu et al., 2001). Thus, we expect that genetic drift and sequence variations in the regions coding for variable surface proteins contribute to an increase in the pan-genome size but that this increase is mainly related to genes encoding proteins without characterized domains.

Because the pan-domainome of mycoplasma species is closed, sequencing additional strains will not add to the overall systems level understanding of mycoplasma physiology and focus should be on further understanding of the mycoplasma strains for which the genome sequence is known. In this study we incorporated the minimal JCVI-Syn3.0, which is based on a *M. mycoides* template. We considered a protein domain essential when it was present in the minimal synthetic bacterium meaning that the protein domain is needed for growth in a complex cultivation medium under laboratory conditions. We also consider it likely that none of the domains in the minimal synthetic bacterium are needed to maintain growth in the specific host since the genome has been minimized for growth outside the host. By comparing the core domainome of the mycoplasma genus with JCVI-Syn3.0 we found that almost all domains present in the mycoplasma core are also present in the minimal synthetic organism and are likely needed to support growth in axenic media under laboratory conditions. The synthetic bacterial genome still contains 17% of essential protein coding genes with an unknown function. We found that conserved hypothetical proteins in the spiroplasma functional group are conserved in JCVI-Syn3.0. This finding is in line with the general notion that conserved hypothetical proteins are more likely to be essential (Galperin and Koonin, 2004) but in the case of mycoplasma this conservation is limited to mainly the functional cluster, and not to the complete genus. Both findings can provide a guideline for the design of minimal bacterial synthetic genomes. We expect that when mycoplasma species from other functional groups are taken as a template, alternative configurations will emerge showing flexibility in the composition of the pan-domainome of minimal synthetic bacteria designed from mycoplasma ancestors.

## Author contributions

All authors contributed to study design and interpretation. TK, JB, and PS drafted the manuscript. JK provided scripts and methods used in this research. All authors revised the manuscript and approved the final version. All authors take responsibility for accuracy and integrity of the work.

## Funding

This work was financially supported by MSD Animal Health, Bioprocess Technology & Support, Boxmeer, Netherlands. This project has received funding from the *European Union's Horizon 2020 research and innovation programme* under grant agreement No. 634942.

### Conflict of interest statement

TK, PV, SJS, and JB are employed by MSD-AH, a pharmaceutical company producing veterinary vaccines. The other authors declare that the research was conducted in the absence of any commercial or financial relationships that could be construed as a potential conflict of interest.
