# Supplementary material for: Persistence of Functional Protein Domains in Mycoplasma Species and their Role in Host Specificity and Synthetic Minimal Life
Source: Front Cell Infect Microbiol. 2017 Feb 7;7:31. doi: 10.3389/fcimb.2017.00031 (PMC5293770; doi:10.3389/fcimb.2017.00031)
Supplement: Supplementary file 2 [file DataSheet2.DOCX]

Supplementary Material

Persistence of Functional Protein Domains in Mycoplasma species and their role in Host Specificity and Synthetic Minimal Life

Tjerko Kamminga^1,2^, Jasper J. Koehorst^1^, Paul Vermeij^3^, Simen-Jan Slagman^2^, Vitor A.P. Martins dos Santos^1^, Jetta J.E. Bijlsma^3^ and Peter J. Schaap^1*^

^1^ Laboratory of Systems and Synthetic Biology, department of Agrotechnology and Food Sciences, Wageningen University and Research, Wageningen, The Netherlands; ^2^ Bioprocess Technology and Support, MSD Animal Health, Boxmeer, The Netherlands; ^3^ Discovery & Technology Research, MSD Animal Health, Boxmeer, The Netherlands

*Corresponding author: [peter.schaap@wur.nl](mailto:peter.schaap@wur.nl)

Contents

[1 Predictions Random Forest classification 3](#_Toc466372711)

[2 Figure S1 6](#_Toc466372712)

[3 Figure S2 7](#_Toc466372713)

[4 Figure S3 8](#_Toc466372714)

[5 Figure S4 9](#_Toc466372715)

[6 Figure S5 10](#_Toc466372716)

# Predictions k-nn and Random Forest classification

*Prediction niche: blood or tissue*

Knn classification for niche: blood or tissue


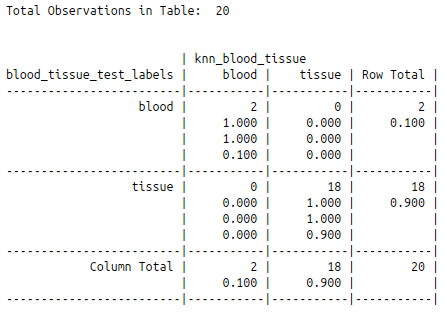


Correct classification of species in the training set (N=20, 2 blood infecting species and 18 tissue infecting species)

Random forest classification for the same dataset

Call:

randomForest(x = domain_table_filtered, y = niche_blood_tissue)

Type of random forest: classification

Number of trees: 500

No. of variables tried at each split: 41

OOB estimate of error rate: 0%

Confusion matrix:

blood tissue class.error

blood 8 0 0

tissue 0 72 0

*Prediction Niche*

Call:

randomForest(x = domain_table_filtered, y = niche)

Type of random forest: classification

Number of trees: 500

No. of variables tried at each split: 41

OOB estimate of error rate: 11.25%

Confusion matrix:

Blood Eyes gils Joints Multiple Respiratory Urogenital class.error

Blood 8 0 0 0 0 0 0 0.00000000

Eyes 0 0 0 0 0 1 0 1.00000000

gils 0 0 0 0 1 0 0 1.00000000

Joints 0 0 0 0 2 0 0 1.00000000

Multiple 0 0 0 0 30 1 0 0.03225806

Respiratory 0 0 0 0 3 33 0 0.08333333

Urogenital 0 0 0 0 1 0 0 1.00000000

*Prediction blood, respiratory or multiple*

Call:

randomForest(x = domain_table_filtered, y = niche4)

Type of random forest: classification

Number of trees: 500

No. of variables tried at each split: 41

OOB estimate of error rate: 5%

Confusion matrix:

Blood Multiple Respiratory class.error

Blood 8 0 0 0.00000000

Multiple 0 36 2 0.05263158

Respiratory 0 2 32 0.05882353

*Selection niche: respiratory or multiple with at least two genomes per species*

Final niche prediction (26 species) ruminant, human, pig with k-nn and random forest:

Niche classification with knn for respiratory/multiple tissue type classification (26 species):


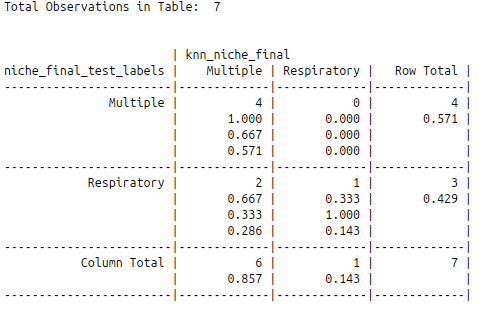


5/7 correct prediction. Poor accuracy for prediction strictly respiratory tissue infection.

Call:

randomForest(x = domain_table_selection_niche4, y = niche_selection4)

Type of random forest: classification

Number of trees: 500

No. of variables tried at each split: 38

OOB estimate of error rate: 3.85%

Confusion matrix:

Multiple Respiratory class.error

Multiple 15 1 0.0625

Respiratory 0 10 0.0000

*Prediction host*

Call:

randomForest(x = domain_table_filtered, y = host)

Type of random forest: classification

Number of trees: 500

No. of variables tried at each split: 41

OOB estimate of error rate: 21.25%

Confusion matrix:

alligator cat chicken cow crocodile dog duck fish goat human pig pigeon rat sheep class.error

alligator 0 0 0 0 1 0 0 0 0 0 0 0 0 0 1.0000000

cat 0 2 0 0 0 0 0 0 0 0 0 0 0 0 0.0000000

chicken 0 0 12 0 0 0 0 0 0 1 1 0 0 0 0.1428571

cow 0 0 0 7 0 0 0 0 0 0 1 0 0 0 0.1250000

crocodile 1 0 0 0 0 0 0 0 0 0 0 0 0 0 1.0000000

dog 0 1 0 0 0 5 0 0 0 0 0 0 0 0 0.1666667

duck 0 0 0 0 0 0 0 0 0 1 0 0 0 0 1.0000000

fish 0 0 0 0 0 0 0 0 0 0 0 0 1 0 1.0000000

goat 0 0 0 1 0 0 0 0 8 0 0 0 0 0 0.1111111

human 0 0 1 0 0 0 0 0 0 15 0 0 1 0 0.1176471

pig 0 0 0 0 0 0 0 0 0 0 14 0 0 0 0.0000000

pigeon 0 0 0 0 0 0 0 0 0 1 0 0 0 0 1.0000000

rat 0 0 0 0 0 0 0 0 0 1 1 0 0 0 1.0000000

sheep 0 0 0 0 0 0 0 0 0 0 3 0 0 0 1.0000000

*Repeat host classification all tissue growing mycoplasma, ruminants used as classification for cow/goat (at least two genomes per species and two species per host)*

k-nn classification


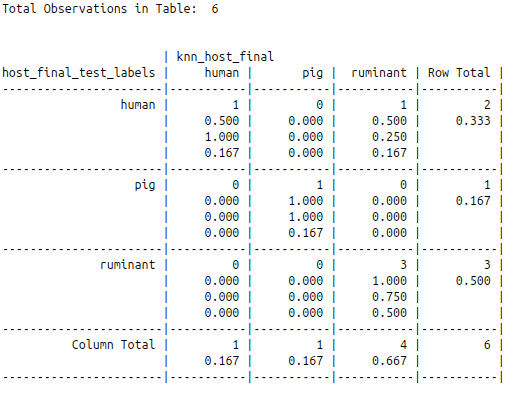


5/6 correctly classified

Random forest classification:

Call:

randomForest(x = domain_table_selection2, y = host_selection2)

Type of random forest: classification

Number of trees: 500

No. of variables tried at each split: 37

OOB estimate of error rate: 0%

Confusion matrix:

human pig ruminant class.error

human 6 0 0 0

pig 0 4 0 0

ruminant 0 0 12 0

# Figure S1

Tissue infecting species

Haemoplasma

Figure S1. Total unique domains in Haemoplasma species (#1-6) and tissue infecting Mycoplasma species.

# Figure S2


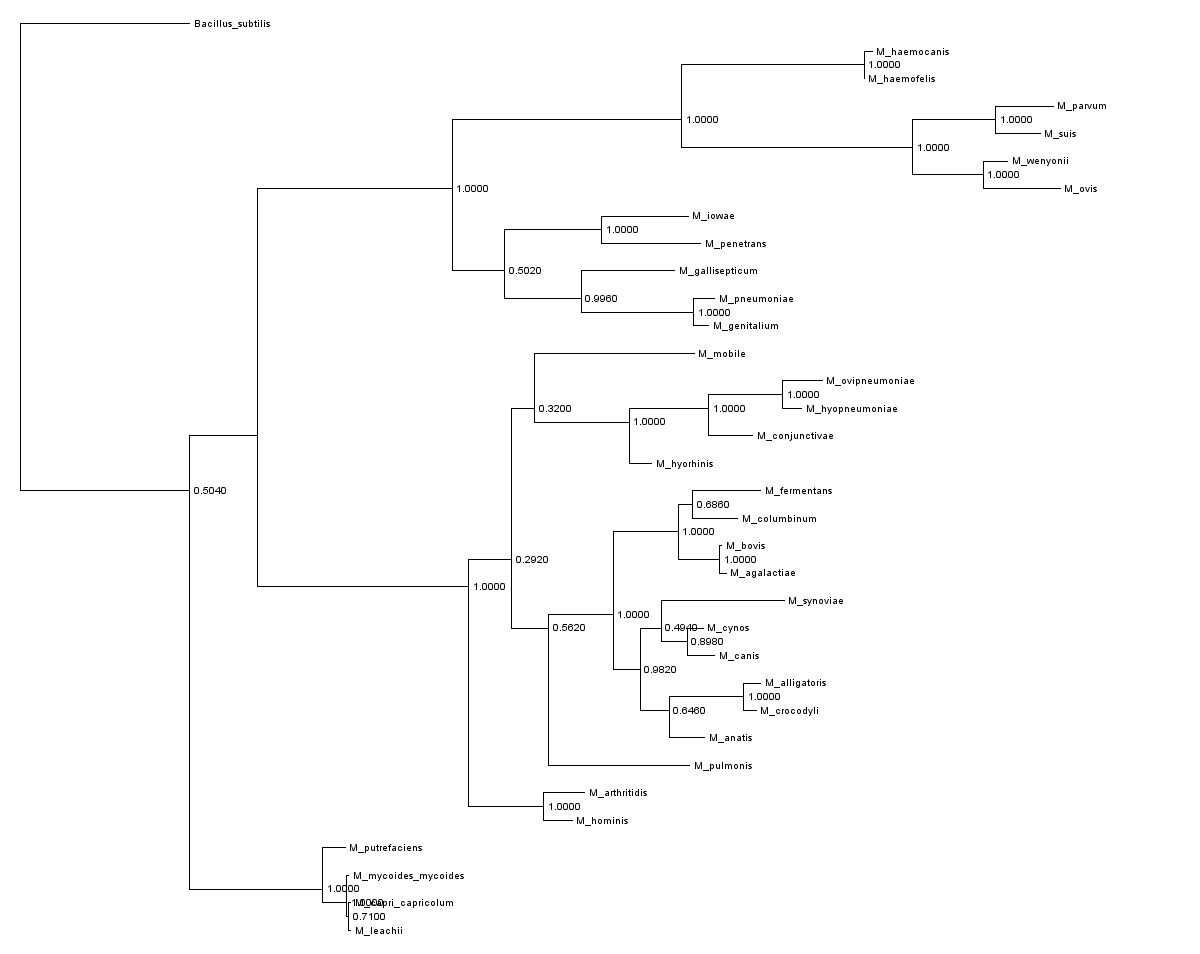


Figure S2. Standard phylogenetic tree using 16S rRNA (maximum likelihood, 500x bootstrapped, see S1 for strains and sequences which were used. Numbers indicate bootstrapping values (percentage by which branch topology was supported).

# Figure S3

Figure S3. Heatmap showing presence (green) or absence (red) of essential domains in JCVI-Syn3.0 not present in the core of the tissue-infecting mycoplasma species.

# Figure S4


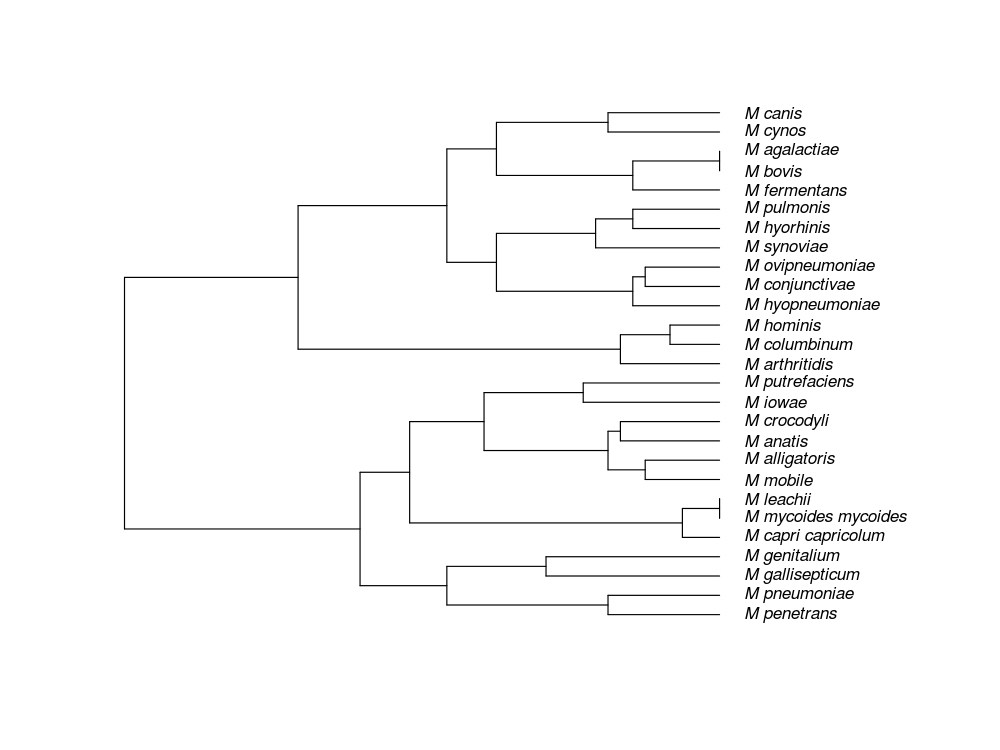


Figure S4. Species clustering on the basis of metabolic domains not essential for minimal life.

# Figure S5


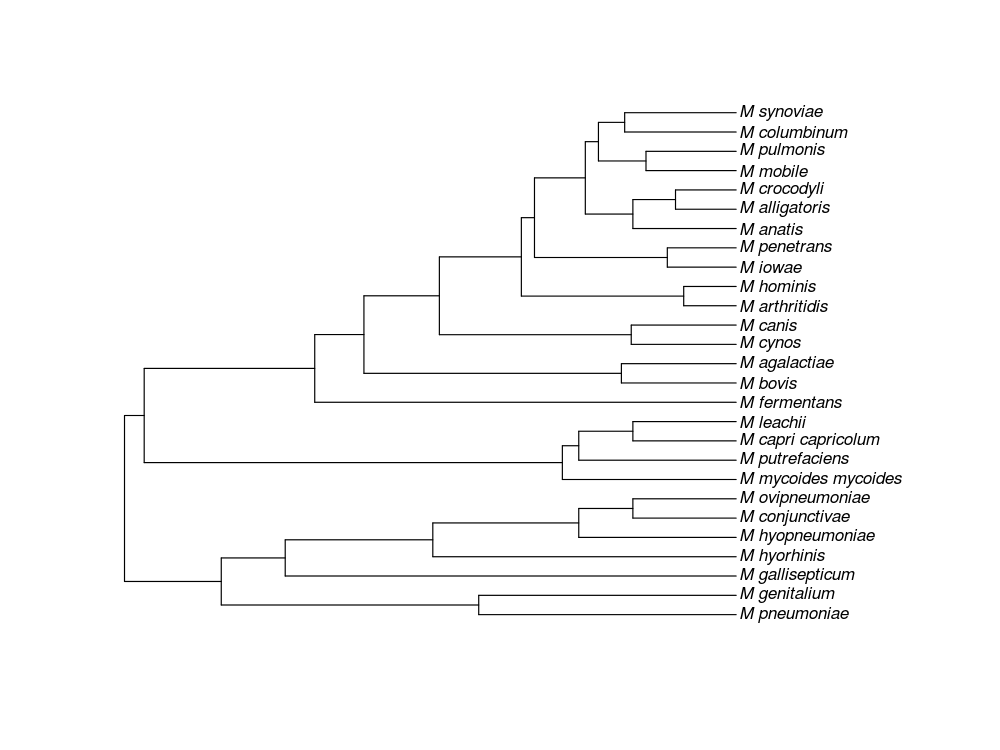


Figure S5. Species clustering on the basis of orthologous hypothetical proteins not essential for minimal life.
